# Pathognomonic

**DOI:** 10.1007/s11606-024-09299-0

**Published:** 2024-12-20

**Authors:** David T. Zhu

**Affiliations:** Medical Scientist Training Program, School of Medicine, Virginia Commonwealth University, Richmond, VA USA

In medical school, we are trained to recognize patterns—certain phrases and pathognomonic signs that stand out like beacons in the sea of symptoms. “Butterfly rash on the face? Lupus.” “Honey-crusted lesions? Impetigo.” These markers of certainty bring comfort in the otherwise uncertain world of illness. But beyond the walls of the hospital and the pages of our textbooks, I find myself wondering: what is pathognomonic of the human condition?

As I reflect on patients with substance use disorders I’ve encountered during my clinical rounds and research, I wonder about their pathognomonic signs. Their experiences and struggles—complex, layered—defy the simplicity of medical flashcards and lecture notes. Addiction cannot be reduced to a neatly packaged set of signs and symptoms; it’s a winding road, one that is shaped by moments of despair, hope, relapse, and recovery. And yet, there is something distinct, something unmistakable about the weight they carry—the exhaustion etched in their faces, the hesitancy in their voices when speaking of their past.

What I’ve come to realize is that the deeper pathognomonic sign of addiction isn’t the stereotypical behaviors written in textbooks but something more universal: disconnection. It’s a separation from relationships, from the self, and from the world. Disconnection is not unique to addiction, but here, it becomes pervasive, permeating every corner of a patient’s life. I often witness it as a profound, almost palpable sense of emotional isolation—a slow unraveling, a slipping away from the person they once knew, from the lives they once imagined. Healing, then, cannot simply be about abstinence. It begins when patients start to rebuild the connections severed by addiction. It happens slowly, through moments of trust—when patients allow themselves, cautiously, to believe in a future they had once feared was lost.

I wonder, then, how to reconcile a deeper meaning of “pathognomonic” beyond its traditional medical ontology. For some patients, it is the silence that hangs heavy with shame and stigma. For others, it’s the unspoken determination in their eyes, a steady flicker of hope that refuses to be extinguished, even in the face of overwhelming odds. Addiction may not present with a singular pathognomonic sign, but there’s a deeper thread running through it—an unspoken fight to reclaim identity, to rediscover hope even after the darkest of days.

The true essence of “pathognomonic” should stretch beyond the physical signs that textbooks train us to recognize. It should encompass the subtle, often invisible markers of a person’s struggle—the weight of their suffering, the resilience in their voice, and the quiet determination to keep moving forward. What is pathognomonic of the human condition is not just what can be named or labeled, but what is felt: the longing for connection, the ache of isolation, and the glimmer of hope that refuses to fade. Perhaps it’s not the signs we can see or touch, but the ones we sense in the fleeting moments when patients allow us into their world. In those moments of shared vulnerability, we encounter the true markers—not of disease, but of what it means to be human.

In the end, perhaps the pathognomonic sign of the human condition, for all of us, is our capacity to endure—to wake up and face the day, to carry on despite everything. And in that persistence, there is a kind of beauty, an intricate balance between resilience and fragility that no one medical term could ever fully define.

